# Shotgun metagenomics reveals the interplay between microbiome diversity and environmental gradients in the first marine protected area in the northern Arabian Gulf

**DOI:** 10.3389/fmicb.2024.1479542

**Published:** 2025-01-09

**Authors:** Saja A. Fakhraldeen, Rakhesh Madhusoodhanan, Nazima Habibi, Sakinah Al-Haddad, Surendraraj Alagarsamy, Sabeena F. K. Habeebullah, Walid M. Al-Zakri, Fathima Thuslim, Loreta Fernandes, Faiza Al-Yamani, Turki Al-Said

**Affiliations:** ^1^Ecosystem-Based Management of Marine Resources Program, Environment and Life Sciences Research Center, Kuwait Institute for Scientific Research, Kuwait City, Kuwait; ^2^Biotechnology Program, Environment and Life Sciences Research Center, Kuwait Institute for Scientific Research, Kuwait City, Kuwait

**Keywords:** microbial diversity, shotgun metagenomics, anthropogenic stressors, hypersaline system, Kuwait Bay, Persian/Arabian Gulf

## Abstract

**Introduction:**

The northwest Arabian Gulf encounters significant anthropogenic pressures, including nutrient enrichment from coastal development and effluent discharge.

**Methods:**

This study presents the first shotgun metagenomics-based characterization of microbial communities in Kuwaiti waters of the northwest Arabian Gulf, focusing on Kuwait’s first Marine Protected Area (MPA) in Sulaibikhat Bay, a vital nursery ground for commercially important fish.

**Results:**

Analysis revealed significantly higher microbial diversity within the MPA compared to adjacent waters, with Rhodobacteraceae (27.8%) and Flavobacteriaceae (15.3%) being dominant. Elevated inorganic phosphorus, nitrogen, and salinity were key factors driving this diversity. Multivariate analysis highlighted phosphate as a critical component affecting the MPA microbial community structure, particularly for the families Microbacteriaceae, Flavobacteriaceae, and Rhodobacteraceae.

**Discussion:**

This study underscores the ecological importance of MPAs and highlights the impact of nutrient enrichment and other environmental stressors on microbial diversity, emphasizing the need to reduce nutrient influx to mitigate eutrophication and enhance marine ecosystem resilience in stressed environments.

## 1 Introduction

The Kuwaiti marine environment in the northwest Arabian Gulf is subjected to extremely harsh conditions due to natural and anthropogenic factors (Al-Mutairi et al., 2014). Environmental natural stressors include extreme temperatures and high salinity, while anthropogenic stressors include land reclamation, urbanization, and desalination activities. Kuwait Bay, a water body located along the eastern coast of Kuwait, is particularly susceptible to these stressors due to its semi-enclosed nature and proximity to heavily urbanized and industrialized areas (Ali and Chidambaram, 2021; Al-Zamel et al., 2009; Butayban and Preston, 2004). All such human activities, combined with the discharge of industrial and residential waste into the Bay, have resulted in the accumulation of high levels of various nutrients and pollutants, leading to the development of highly eutrophic conditions (Alsabti et al., 2022; Al-Sarawi et al., 2015; Gevao et al., 2021; Habibi et al., 2021).

Exposure to extreme environmental conditions and anthropogenic activities often necessitates the development of functional genetic adaptations that enable resident organisms to survive in these harsh conditions. Globally, such adaptations have been documented in a wide range of organisms, including fish, macroalgae, plankton, and bacteria (Yooseph et al., 2010; Parrilli et al., 2021; Wani et al., 2022; Chiriac et al., 2023; Tilahun et al., 2024; Shu and Huang, 2022). In Kuwait, for instance, one study investigated the effect of seasonality on heat shock protein (HSP) expression levels in fish from Kuwait Bay (Beg et al., 2010). Additionally, altered gene expression has been observed in different generations of copepods exposed to elevated temperatures and lowered pH (Habibi et al., 2023b).

Kuwait Bay is home to several commercially important species of fish, shrimp, and other invertebrates. Therefore, it is crucial to protect this waterbody from the detrimental impacts of environmental extremes and anthropogenic activities not only to protect biodiversity, but also to preserve economic interests. Microorganisms play a vital role in maintaining ecosystem biological productivity and are among the most significantly affected by environmental and anthropogenic stressors (Dang et al., 2019; Abirami et al., 2021; Crowther et al., 2024; Nelson et al., 2023; Osburn et al., 2023). Previous studies have documented the structure and diversity of bacteria, archaea, and other microbial communities within Kuwaiti waters and examined their spatiotemporal variability in the water column (Fakhraldeen et al., 2023; Chen, 2017; Kumar et al., 2021; Al-Rifaie et al., 2008). Studies have also investigated the effects of various physicochemical parameters including salinity, seawater temperature, and dissolved oxygen levels, on the microbiome within these waters (Fakhraldeen et al., 2023; Al-Said et al., 2017). Overall, these studies demonstrated that the Kuwaiti marine microbiome is a rich and dynamic community of microorganisms (Habibi et al., 2023a).

One of the most biologically productive areas within Kuwait Bay is Sulaibikhat Bay, a shallow tide-dominated embayment. Recently designated as a marine protected area (MPA) by the United Nations Compensation Commission, extensive efforts are underway to characterize the physical, chemical, and biological properties of this water body. This study aimed to characterize the microbiome of the MPA in Sulaibikhat Bay and, importantly, to compare the MPA microbiome with those of surrounding waters that are exposed to differing levels of anthropogenic stressors.

Surface seawater samples were collected for four consecutive months from four sampling locations in Kuwaiti waters: the MPA in Sulaibikhat Bay (St. MPA-2), Kuwait Bay (St. K6), northern coastal waters (St. A), and southern offshore waters (St. 18). The bacterial and archaeal communities at these locations were studied using a shotgun metagenomics approach. Subsequently, the microbiome at each of the four sites was analyzed with respect to nitrogenous and non-nitrogenous nutrient levels in the seawater at the time of sampling. The results from this study offer insights into the effect of anthropogenic influence and various nutrients on the microbiome in Kuwaiti waters.

## 2 Materials and methods

### 2.1 Sampling site description and sample acquisition

**FIGURE 1:**
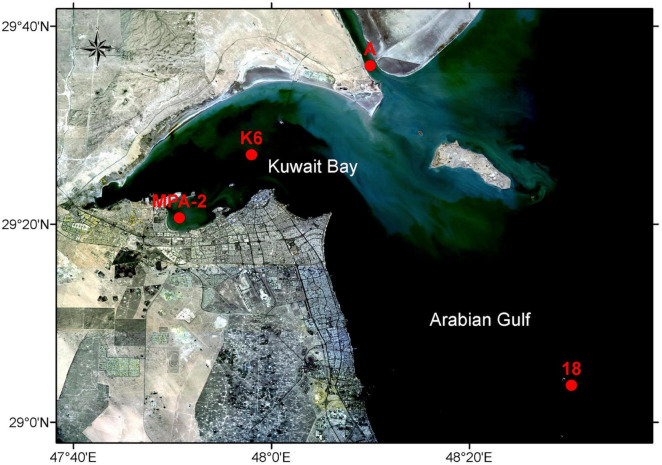
Sample collection sites. Red dots indicate the sites from which seawater samples were collected for this study (St. MPA-2 located in the MPA in Sulaibikhat Bay, St. K6 located inside Kuwait Bay, St. A located in northern coastal waters to the northeast of Kuwait Bay, and St. 18 located in southern offshore waters to the southeast of Kuwait Bay). Basemap was obtained from Landsat (http://landsat.visibleearth.nasa.gov/) and annotated using the ArcGIS software (https://www.arcgis.com/). Figure courtesy of T. Yamamoto, Kuwait Institute for Scientific Research.

Surface seawater samples were collected from four stations: St. MPA-2, located in the MPA in Sulaibikhat Bay; St. K6, located in Kuwait Bay; St. A, located in northern coastal waters to the northeast of Kuwait Bay; and St. 18, located in southern offshore waters to the southeast of Kuwait Bay (Figure 1 and Table 1). The sampling was conducted for four consecutive months (November and December 2019, and January and February 2020). Samples were collected using 5 L Niskin bottles (General Oceanics, Florida, USA) and stored in sterilized 5 L polypropylene carboys (Azlon cat# BNP05B) for nucleic acid extraction and shotgun metagenomic sequencing. Seawater samples for the analysis of dissolved inorganic nutrients such as nitrate (NO_3_^–^), nitrite (NO_2_^–^), phosphate (PO_4_^3^), and silicic acid (SiO_4_^4–^) were transferred into 1 L acid-washed polyethylene bottles for laboratory processing. Seawater samples for ammonium (NH_4_) measurements were filtered using syringe filters with 25 mm 0.7 μm glass fiber filters (GF/F) and transferred into clean 125 ml polyethylene bottles. The sample volume was then divided into a triplicate set of 10 ml each in 14 ml test tubes with airtight caps. Phenol and hypochlorite reagents were added in the field. A total of 6 samples were collected from St. MPA-2, and a total of 4 samples were collected from each of St. K6, A, and 18. Complete sampling details including GPS coordinates, time, date, etc. are provided in Supplementary Table 1.

**TABLE 1:** Sampling site details (location, average sampling depth, seawater temperature, salinity, and dissolved oxygen levels) for the 4 months of sampling.

Station	GPS coordinates*	Average station depth (m)	Average sample acquisition depth (m)	Average seawater temperature (°C)	Average salinity	Average dissolved oxygen (ml/L)
St. MPA-2	29° 20.629 N 45° 50.721 E	1.8	1.0	20.2	42.3	7.4
St. K6	29° 26.987 N 47° 58.009 E	10.7	1.3	19.2	40.8	7.6
St. A	29° 35.995 N 48° 09.997 E	6.5	1.3	17.8	39.1	7.8
St. 18	29° 03.727 N 48° 30.336 E	20.6	1.3	22.1	40.6	6.8

*GPS coordinates listed are from November 2019 sampling. There were no significant variations in the GPS coordinates registered during each sampling excursion. A complete list of GPS coordinates is provided in Supplementary Table 1.

### 2.2 Collection of metadata

Data describing different physicochemical aspects of the environment at the time of sample acquisition was previously described (Fakhraldeen et al., 2023). The environmental parameters measured were total sampling location depth (m), dissolved O_2_ levels (ml/L), salinity, and seawater temperature (°C), all of which were measured using a portable water quality profiler equipped with pre-calibrated sensors (AAQ-RINKO, JFE Advantech, Hyogo, Japan). Wave height (m) was measured using pressure-based wave and tide acoustic sensors and sea color was assessed using the Forel and Ule scales. The measured parameters related to air were air temperature in shade (°C), wind direction, wind speed (kph), relative humidity (RH%), and barometric/atmospheric pressure (hectopascals), all of which were assessed/measured using a portable weather station (Kestrel, Pennsylvania, USA). A visual assessment of cloud cover was also made. All measurements are tabulated in Supplementary Table 2.

### 2.3 Measurement of dissolved nutrient levels

Seawater samples for nutrient analysis were filtered through 0.7 μm glass fiber filters (GF/F, Whatman, Maidstone, UK), transferred to 125 ml acid-washed polyethylene bottles, and stored at −20°C until analysis. A colorimetric-based method was used to measure the amount of nutrients (excluding ammonium) in the samples using an automated analyzer (Skalar San ++, Breda, The Netherlands) (ROPME, 2010). A combination of the Sagi and Solorzano methods (Sagi, 1966; Solorzano, 1969) was applied to measure ammonium levels. Optical density measurements of the levels of indophenol blue that is produced when ammonium reacts with the Berthelot reagent (containing phenol and hypochlorite) at a wavelength of 630 nm using a double-beam scanning spectrophotometer were performed (Shimadzu UV-2600, Kyoto, Japan). Triplicate measurements were carried out for each nutrient in each sample.

### 2.4 DNA extraction and library preparation

DNA isolation from seawater samples was performed as previously described (Fakhraldeen et al., 2023). Approximately 1 L of each sample was subjected to vacuum filtration through a 0.22 μm polycarbonate membrane (Isopore/Millipore cat# GTTP040700, Darmstadt, Germany). Membranes were then cut using sterile razor blades, and the cut membrane pieces were used as the starting material for DNA isolation. DNA extraction was carried out using the PowerSoil^®^ DNA Isolation Kit (Qiagen, Hilden, Germany) as per the manufacturer’s instructions. DNA yield assessment was performed using a NanoDrop (Thermo Fisher Scientific, Massachusetts, USA). The quality of the extracted DNA was also assessed through gel electrophoresis. The eluted DNA was run on a 1% agarose gel containing 10 mg/ml ethidium bromide at 100 V for 60 min. Gels were then imaged using the ChemiDoc MP gel documentation system (Bio-Rad, California, USA) to ensure the absence of sheared DNA. The average 260/280 ratio measured by Nanodrop was 1.965, and all bands detected on agarose gels were sharp and intact with little to no smearing observed.

### 2.5 Shotgun metagenomic sequencing and data processing

Library preparation, DNA sequencing, and data processing were performed as described in Fakhraldeen et al. (2023). A total of 1 ng of DNA was processed using the Nextera^§^ XT DNA Library Preparation Kit (Illumina, California, USA). Library quantity and quality were assessed using the Qubit 2.0 system (Thermo Fisher Scientific, Massachusetts, USA) and the Tape Station 2200 system coupled with high sensitivity D1000 Screen Tapes (Agilent Technologies, Inc., California, USA), respectively. Libraries were pooled in equimolar amounts based on quality control values and then sequenced using the HiSeq platform (Illumina, California, USA) to generate 2 × 150 bp reads at a depth of 20 million paired-end reads per sample (10 million in each direction). Trimmomatic (version v0.38) (Bolger et al., 2014) was then used to cut adapter sequences and trim low-quality bases with the following settings: phred score 33, SLIDINGWINDOW of 4:20, LEADING and TRAILING threshold of 20, and drop if length <100 (MNLEN 100). FastQC (version v0.11.8)^1^ was applied to check the quality of raw reads. MEGAHIT (v1.2.9) NGS assembler (Li et al., 2015; Li et al., 2016) was used to assemble the trimmed fastq data and generate assembled contigs of average length 642 bp. Average contig lengths generated by MEGAHIT per sample are provided in Supplementary Table 2.

### 2.6 Bacterial and archaeal microbiome analysis

MetaPhlAn (v3.0) was used to profile the composition of microbial communities (Segata et al., 2012). MetaPhlAn’s latest database files were downloaded from http://cmprod1.cibio.unitn.it/biobakery3/metaphlan_databases/. The most recent mpa file for MetaPhlAn3 is mpa_v30_CHOCOPhlAn_201901. This file was used with the “– index” parameter. The trimmed reads generated by MEGAHIT were used as inputs for MetaPhlAn3 to increase the sensitivity of the output. Default values were kept for all other parameters. “Very-sensitive” mode was used when aligning reads. The minimal mapping quality was set to 5. The minimum total nucleotide length for the markers in a clade for estimating the abundance without considering sub-clade abundances was set to 2000. Additionally, the threshold for the percentage of markers with a non-zero relative abundance for misidentified species was established at 33%.

The R package metacoder (v0.3.4) was used to generate the heat trees (Foster et al., 2017). The node size and color are mapped relative to the observed read numbers and Operational Taxonomic Unit (OTU) counts, respectively.

The R package “microbiome 1.13.10” (Lahti and Shetty, 2018) was used to analyze the core microbiome at the family, genus, and species levels. The “plot_core” function was used to generate the core microbiome figure. The minimum prevalence was set to 0.1 and applied to the rows (taxa) and the columns (detections).

### 2.7 Alpha and beta diversity analyses

Alpha diversity, represented by four indices (Chao1, Shannon, Simpson, and InvSimpson), was estimated using the R package phyloseq (v1.34.0) (McMurdie and Holmes, 2013). The alpha diversity was compared between each pair of stations (St. MPA-2 (MPA in Sulaibikhat Bay) versus St. K6 (Kuwait Bay), St. MPA-2 versus St. A, and St. MPA-2 versus St. 18) through One Way Analysis of Variance (ANOVA) (*p* < 0.05).

Principal component analysis (PCA) was performed using the R function prcomp in R package stats4 (v4.0.5)^2^ using singular value decomposition of the centered and scaled data matrix (taxonomy count table). The principal components (PC1, 2, and 3) from the samples are shown on 3D scatter plots generated using scatterplot3d (v0.3-41). The proportion of variance is also depicted on the plots.

Differential heat trees were generated using the same tool and approach described above, except for the node color being mapped to the log 2 median ratio of abundance values (OTU counts) between various pairs of samples instead of the raw OTU counts.

Analysis of Similarity (ANOSIM) was performed using the R package “anosim” in the package vegan (v.2.5-7) (Oksanen, 2010). Differential abundance analysis was performed using the Metastats tool (White et al., 2009). The tool outputs the mean value, variance, standard deviation, *p*-value, and *q*-value. The top differentially abundant taxa are displayed graphically.

### 2.8 Multivariate analysis of environmental and biological data sets

The biotic and environmental datasets were initially screened using the D’Agostino-Pearson Omnibus Normality Test. As the test suggested a non-normal distribution of data points, the datasets were processed using non-parametric statistical methods. In the environmental metadata, water temperature, salinity, and dissolved oxygen datasets showed no distribution skewness. Hence, their values were not transformed. Values of all nutrient variables and turbidity levels were individually transformed by taking the square root of each value. The selectively transformed and untransformed variables were standardized before constructing the Euclidean distance-based similarity matrix required for ordination analyses. The bacterial OTU abundance values were transformed by taking the natural log of each value in the biological dataset before constructing the Bray-Curtis dissimilarity matrix. The dominance and diversity of bacterial taxa (at different taxonomic levels) at different sampling areas were investigated through cumulative k-dominance plots. The constraint ordination procedure, Canonical Analysis of Principal Coordinates (CAP), was used to explore the microbial assemblage patterns at the species, genus, and family levels. Before the CAP analysis, the sample clustering patterns were probed through the Similarity Profile (SIMPROF) analysis and its *Pi* statistics. The Permutational Multivariate Analysis of Variance (PERMANOVA) explored the differences in microbiome structure between the sampling locations and the significance of clustering patterns through its pseudo-t-statistics and associated permutational and Monte Carlo-based significance tests. The linear combinations of environmental variables bearing correlation of >0.2 (Pearson) with the biotic assemblages and CAP axes were overlaid as vectors on the CAP plot. Additionally, 3D bubble plots were constructed to show the distribution of environmental variables across the sample groups. A colored, interactive heatmap with XY clustering for samples and species explored the sample clustering based on bacterial species association patterns. A Venn diagram was used to explore the species turnover (i.e., beta diversity) between the sample groups and to enlist the endemic and common species. The relationship between biotic and environmental rank matrices was explored using RELATE, a Mantel test based on Spearman rank correlation. The multivariate Biotic-Environmental interactions procedure (BIOENV) was used to find the best combination of environmental variables responsible for the bacterial assemblage patterns.

All multivariate analyses were performed in the ecological statistical software PRIMER v7.0.11 (PRIMER E-Ltd., UK) (Clarke and Gorley, 2015) with PERMANOVA + 1 add-on for PRIMER (Anderson, 2008). The Venn diagram was constructed using the freely available Bioinformatics & Evolutionary Genomics software.^3^

## 3 Results

### 3.1 Metagenomic sequencing and assembly

Libraries were used to generate paired-end sequences (150 × 2) resulting in an average of 28.3 million reads total and an average of 14.2 million paired-end reads per sample (Phred > Q20 for 97% of reads). Reads were subjected to quality filtering and trimming resulting in the removal of 1.3% of reads. The retained reads yielded approximately 27.97 million clean, processed reads per sample for downstream analysis. Reads from each sample were assembled *de novo* into scafftigs with the largest scafftigs ranging in length from 37,426 to 276,400 bp. The N50 values of the assembled genomes ranged from 571 to 1024. Detailed read statistics are available in Supplementary Table 3.

### 3.2 Environmental conditions at the sampling locations

A number of physicochemical parameters were assessed at the time of sample acquisition from the four different sampling locations throughout the study period (Supplementary Table 4). The depth of the stations was logged, and measurements showed that the sampling locations in order of increasing average depth were St. MPA-2 (1.8 m) < St. A (6.5 m) < St. K6 (10.7 m) < St. 18 (20.6 m). Average surface seawater sample acquisition depth was 1.0 m for St. MPA-2 and 1.3 m for the other three stations. Dissolved oxygen (DO_2_) levels were also measured and showed no significant difference between the sampling stations throughout the study period. Average DO_2_ levels, in increasing order, were recorded at St. 18 (6.8 mg/l) < St. K6 (7.6 mg/l) < St. A and St. MPA-2 (both 7.8 mg/l).

Significant differences were recorded, however, in seawater temperature and salinity levels between the stations. The highest average seawater temperature was recorded at St. 18 (22.1°C), which was significantly greater than the average temperatures recorded at both Stations A (17.8°C; *p* = 0.012) and K6 (19.2°C; *p* = 0.013). Average temperatures recorded at St. MPA-2 (19.6°C) was not significantly different from the other stations (*p* = 0.241, 0.795, and 0.242 for Stations A, K6, and 18, respectively; Student’s *t*-test). Stations K6, A, and 18 all had similar salinity levels ranging from 39.1 at St. A to 40.8 at St. K6. Salinity levels at St. MPA-2 were significantly higher, though, at 42.4 (*p* < 0.03).

Overall, St. MPA-2 in Sulaibikhat Bay was the shallowest of the four stations examined and had the highest average salinity levels. The offshore station located to the southeast of Kuwait Bay, St. 18, had the highest recorded average seawater temperature compared to the other three stations. Dissolved oxygen levels were similar between all four stations throughout the study period.

### 3.3 Taxonomic profiling of bacterial and archaeal communities in the MPA in Sulaibikhat Bay and assessment of the core microbiome

**FIGURE 2:**
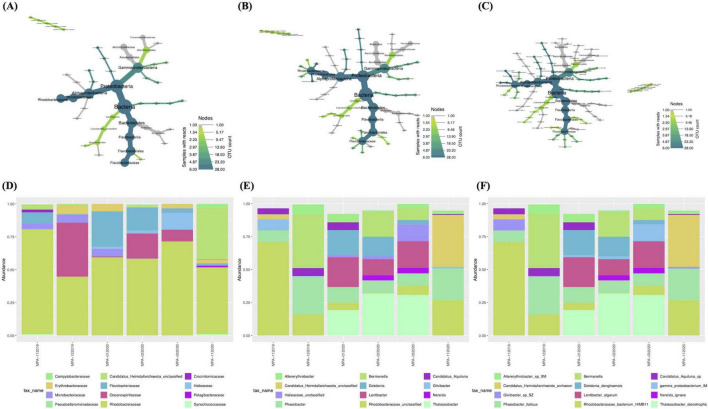
Taxonomic profiling of bacterial and archaeal communities in St. MPA-2 in Sulaibikhat Bay. Bacterial and archaeal community composition in St. MPA-2 during the period between November 2019-February 2020 are depicted in the form of heat trees at the **(A)** family, **(B)** genus, and **(C)** species levels and in the form of stacked bar plots at the **(D)** family, **(E)** genus, and **(F)** species levels. The size and color of the nodes in the heat trees are shown relative to the read numbers and OTU counts, respectively. The twelve most abundant taxonomic groups per classification are shown in the stacked bar plots.

Taxonomic profiling was performed on the six samples collected from St. MPA-2 in Sulaibikhat Bay to generate a profile of the bacterial and archaeal communities within that water body. Depictions of the twelve most abundant taxa at the family, genus, and species levels are provided (Figure 2).

Results documented 18 families, including 17 bacterial families and one archaeal family, with relative abundances (RA%) of 92.78 and 7.22%, respectively. Of the bacterial families, 50% (9/18) belonged to the phylum Proteobacteria, 22% (4/18) belonged to the phylum Bacteroidota, 11% (2/18) belonged to the phylum Cyanobacteria, and then one family each belonged to the phyla Actinomycetota and Campylobacterota. The Proteobacteria phylum was represented by two classes, Gammaproteobacteria (5/9) and Alphaproteobacteria (4/9). The phylum Bacteroidota was represented equally by the two classes, Flavobacteriia and Bacteroidia. Furthermore, the family *Rhodobacteraceae* (class Alphaproteobacteria) had the highest RA (60.64%), followed by the families *Oceanospirillaceae* (class Gammaproteobacteria; 11.66%) > *Flavobacteriaceae* (class Flavobacteriia; 9.67%) > *Erythrobacteraceae* (class Alphaproteobacteria; 3.24%) > *Microbacteriaceae* (class Actinobacteria; 2.84%) > *Halieaceae* (class Gammaproteobacteria; 2.74%). The families *Campylobacteraceae*, *Crocinitomicaceae*, *Synechococcaceae*, and *Pelagibacteraceae* all had RAs between 0.2 and 1% while the remaining families all had RAs < 0.1% (Figures 2A, D and Supplementary Table 5).

A total of 26 genera were detected. The two families with the most genera were *Rhodobacteraceae* [five genera in decreasing order of RA *Rhodobacteraceae_unclassified* (21.09%) > *Phaeobacter* (15.4%) > *Thalassobacter* (13.62%) > *Lentibacter* (9.16%) > *Nereida* (1.37%)] and *Flavobacteriaceae* (five genera in decreasing order of RA: *Dokdonia* (6.21%) > *Gilvibacter* (1.68%) > *Polaribacter* (1.19%) > *Formosa* (0.58%) > *Cloacibacterium* (0.01%). All other families were represented by just one genus each. The five most abundant genera from this latter category were *Bermanella* (11.66%), *Candidatus_Heimdallarchaeota_unclassified* (7.22%), *Altererythrobacter* (3.24%), *Candidatus_Aquiluna* (2.84%), and *Halieaceae_unclassified* (2.74%) from the families *Oceanospirillaceae*, *Candidatus_Heimdallarchaeota_unclassified*, *Erythrobacteraceae*, *Microbacteriaceae*, and *Halieaceae*, respectively (Figures 2B, E and Supplementary Table 5).

A total of 29 species were documented. The sole archaeal species was *Candidatus_Heimdallarchaeota_archaeon* belonging to the phylum Candidatus_Heimdallarchaeota and an unclassified family. The ten most abundant bacterial species in decreasing order of abundance were *Rhodobacteraceae_bacterium_HIMB11* (21.09%) > *Phaeobacter_italicus* (15.4%) > *Thalassobacter_ stenotrophicus* (13.62%) > *Bermanella_sp* (11.66%) > *Lentibacter_algarum* (9.16%) > *Dokdonia_donghaensis* (6.21%) > *Altererythrobacter_sp_XM_24bin4* (3.24%) > *Candidatus_ Aquiluna_sp_XM_24bin5* (2.84%) > *Gammaproteo bacterium_IMCC3088* (2.74%) > *Gilvibacter_sp_SZ_19* (1.68%) (Figures 2C, F and Supplementary Table 5).

The core microbiome within the six samples collected from St. MPA-2 in Sulaibikhat Bay was assessed. Results showed that the 12 dominant families included *Rhodobacteraceae* (represented by five genera collectively accounting for an overall prevalence of 27.8%) and *Flavobacteriaceae* (represented by four genera collectively accounting for an overall prevalence of 15.3%). The following families were represented by one genus each unless otherwise stated: *Erythrobacteraceae* (6.94%), *Campylobacteraceae* (6.94%), *Microbacteriaceae* (5.56%), *Oceanospirillaceae* (5.56%), *Halieaceae* (4.17%), *Pseudoalteromonadaceae* (represented by two genera collectively accounting for an overall prevalence of 2.8%), an unclassified archaeal family (2.78%), *Synechococcaceae* (2.78), *Crocinitomicaceae* (2.78%), *Pelagibacteraceae* (1.39%), and *Bacteroidaceae* (1.39%) (Supplementary Figure 1A and Supplementary Table 6).

The 12 most prevalent genera in descending order of abundance were *Phaeobacter* (8.33%), *Rhodobacteraceae_unclassified* (8.33%), *Altererythrobacter* (6.94%), *Arcobacter* (6.94%), *Bermanella* (5.56%), *Candidatus_Aquiluna* (5.56%), *Thalassobacter* (4.17%), *Lentibacter* (4.17%), *Dokdonia* (4.17%), *Halieaceae_unclassified* (4.17%), *Gilvibacter* (4.17%), and an unclassified archaeal genera *Heimdallarchaeota_unclassified* (2.78%) (Supplementary Figure 1B).

31 species were detected at a prevalence above 0.1. The top 12 species represented in the core microbiome were *Phaeobacter_italicus* (8.33%), *Rhodobacteraceae _bacterium_HIMB11* (8.33%), *Altererythrobacter_sp_XM_24bin4* (6.94%), *Bermanella_sp* (5.56%), *Thalassobacter_stenotrophicus* (4.17%), *Lentibacter_algarum* (4.17%), *Candidatus_Aquiluna _sp_XM_24bin5* (5.56%), *Dokdonia_donghaensis* (4.17%), *Arco bacter_cryaerophilus* (6.94%), *Gamma_proteobacterium_IMCC3088* (4.17%), *Gilvibacter_sp_SZ_19* (4.17%), and *Candidatus_Heimdallarchaeota_archaeon* (2.78%) (Supplementary Figure 1C).

### 3.4 Comparison of the taxonomic profiles of bacterial and archaeal communities between the MPA in Sulaibikhat Bay and surrounding waters

#### 3.4.1 Station K6 (Kuwait Bay)

**FIGURE 3:**
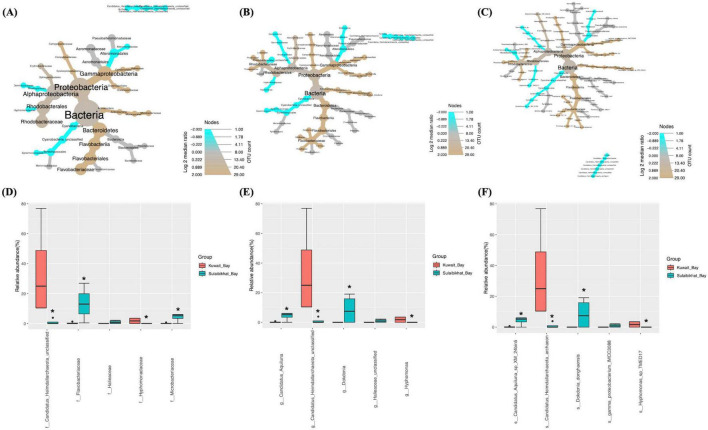
Comparison of microbial abundances between St. MPA-2 in Sulaibikhat Bay and Kuwait Bay (St. K6). Differential heat trees were generated at the **(A)** family, **(B)** genus, and **(C)** species levels. The size and color of the nodes are mapped relative to the observed read numbers and the log 2 median ratio, respectively. Differential abundance analysis was performed at the **(D)** family, **(E)** genus, and **(F)** species levels comparing St. MPA-2 in Sulaibikhat Bay (blue) with Kuwait Bay (St. K6; red). *q < 1; *p* < 0.04 (based on non-parametric *t*-test).

The bacterial and archaeal abundances in St. MPA-2 in Sulaibikhat Bay and St. K6 in Kuwait Bay were compared through differential testing. Results revealed that one bacterial order, one bacterial family, two bacterial genera, and two bacterial species were significantly differentially abundant between St. MPA-2 and St. K6. The bacterial order Rhodospirillales was significantly (*p* = 0.0265) preferentially abundant in St. K6 compared to St. MPA-2. Within this order, the family *Geminicoccaceae*, genus *Geminicoccus*, and species *Geminicoccus_sp* were significantly (*p* = 0.0265) more abundant in St. K6 compared to St. MPA-2. However, another species, *Phaeobacter_italicus* exhibited significantly (*p* = 0.021) higher abundance in St. MPA-2 compared to St. K6 (Figures 3A–C and Supplementary Table 7). Overall, results showed no significant difference (*p* = 0.124) in the total abundance of bacterial versus archaeal communities between the two stations. Differential abundance analysis revealed four of 27 families were significantly differentially abundant between St. MPA-2 and St. K6. The bacterial families *Microbacteriaceae* (*p* = 0.003) and *Flavobacteriaceae* (*p* = 0.0175) were significantly more abundant at St. MPA-2 while the archaeal family *Candidatus_Heimdallarchaeota_unclassified* (*p* = 0.0263) and the bacterial family *Hyphomonadaceae* (*p* = 0.04) were significantly more abundant at St. K6 (Figure 3D and Supplementary Table 8). Four out of 29 genera detected at the genus level showed significantly differential abundance between the two stations. Specifically, the bacterial genera *Candidatus_Aquiluna* (*p* = 0.007) and *Dokdonia* (*p* = 0.04) were significantly more abundant at St. MPA-2 while the archaeal genus *Candidatus_Heimdallarchaeota_unclassified* (*p* = 0.02) and the bacterial genus *Hyphomonas* (*p* = 0.027) showed significantly greater abundance at St. K6 (Figure 3E and Supplementary Table 8). Finally, four out of 32 species detected showed significantly differential abundance between the two stations. Specifically, the bacterial species *Candidatus_Aquiluna_sp_XM_24bin5* (*p* = 0.005) and *Dokdonia_donghaensis* (*p* = 0.039) were significantly more abundant at St. MPA-2, while the archaeal species *Candidatus_Heimdallarchaeota_archaeon* (*p* = 0.017) and the bacterial species *Hyphomonas_sp_TMED17* (*p* = 0.025) showed significantly greater abundance at St. K6 (Figure 3F and Supplementary Table 8).

#### 3.4.2 Station A (northern coastal waters)

**FIGURE 4:**
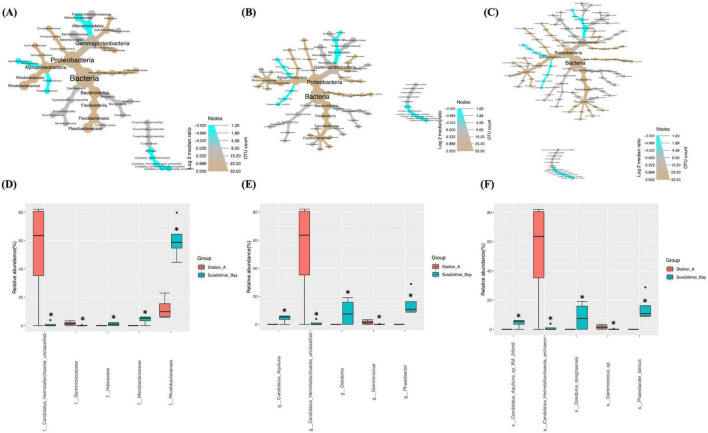
Comparison of microbial abundances between St. MPA-2 in Sulaibikhat Bay and northern coastal waters (St. A). Differential heat trees were generated at the **(A)** family, **(B)** genus, and **(C)** species levels. The size and color of the nodes are mapped relative to the observed read numbers and the log 2 median ratio, respectively. Differential abundance analysis was performed at the **(D)** family, **(E)** genus, and **(F)** species levels comparing St. MPA-2 in Sulaibikhat Bay (blue) with northern coastal waters (St. A; red). *q ≤ 0.81; *p* ≤ 0.04 (based on non-parametric *t*-test).

Comparative analysis of bacterial and archaeal abundances between St. MPA-2 and St. A showed that, of the six bacterial phyla documented, only one exhibited significantly (*p* = 0.029) differential abundance between these locations. The abundance of the phylum Proteobacteria and the Class Alphaproteobacteria were significantly (*p* = 0.029) higher in St. MPA-2 in Sulaibikhat Bay compared to St. A. At the lower taxonomic levels, abundances of two out of the 15 orders detected were significantly different between these locations. The abundance of the order Pelagibacterales was significantly higher at St. A (*p* = 0.021) whereas abundance of the order Rhodobacterales was significantly higher at St. MPA-2 (*p* = 0.029). Both these orders belong to the class Alphaproteobacteria. Three families *Alteromonadaceae* (*p* = 0.0265), *Pelagibacteraceae* (*p* = 0.021), and *Rhodobacteraceae* (*p* = 0.029) were significantly differentially abundant between the two stations with the former two being significantly more abundant at St. A, and the latter one showing significantly greater abundance at St. MPA-2 (Figures 4A–C and Supplementary Table 7) Three of the 35 species detected showed significantly differential abundance between St. MPA-2 and St. A. The abundance of the species *alpha_proteobacterium_HIMB114* (*p* = 0.021) and *Alteromonas_macleodii* (*p* = 0.0265) belonging to an unclassified genus in the family *Pelagibacteraceae* and the genus *Alteromonas*, respectively was significantly higher at St. A. The species *Phaeobacter_italicus* (*p* = 0.021) showed significantly greater abundance at St. MPA-2 (Figures 4A–C and Supplementary Table 7).

Differential abundance analysis revealed that five of the 27 families documented were significantly differentially abundant between St. MPA-2 and St. A. Specifically, the bacterial families *Rhodobacteraceae* (*p* = 0), *Microbacteriaceae* (*p* = 0.003), and *Halieaceae* (*p* = 0.04) were significantly more abundant in St. MPA-2 compared to St. A, whereas the archaeal family *Candidatus_Heimdallarchaeota_unclassified* (*p* = 0.017) and the bacterial family *Geminicoccaceae* (*p* = 0.028) were significantly more abundant in St. A (Figure 4D and Supplementary Table 9). Six of the 30 genera detected showed significant differential abundance between the two stations. Namely, the bacterial genera *Phaeobacter* (*p* = 0.0009), *Candidatus_Aquiluna* (*p* = 0.0017), *Dokdonia* (*p* = 0.026), and *Halieaceae_unclassified* (*p* = 0.04) were significantly more abundant in St. MPA-2 whereas the archaeal genus *Candidatus_Heimdallarchaeota_unclassified* (*p* = 0.01) and the bacterial genus *Geminicoccus* (*p* = 0.02) were significantly more abundant in St. A (Figure 4E and Supplementary Table 9). Finally, the abundances of six of the 36 species detected showed a significant difference between the two stations. The bacterial species *Phaeobacter_italicus* (*p* = 0.0009), *Candidatus_Aquiluna_sp_XM_224bin5* (*p* = 0.002), *Dokdonia_donghaensis* (*p* = 0.021), and *Gamma_proteobacterium_IMCC3088* (*p* = 0.033) were significantly more abundant in St. MPA-2 whereas abundance of the archaeal genus *Candidatus_Heimdallarchaeota_archaeon* (*p* = 0.009) and the bacterial genus *Geminicoccus_sp* (*p* = 0.018) were significantly higher in St. A (Figure 4F and Supplementary Table 9).

#### 3.4.3 Station 18 (southern offshore waters)

**FIGURE 5:**
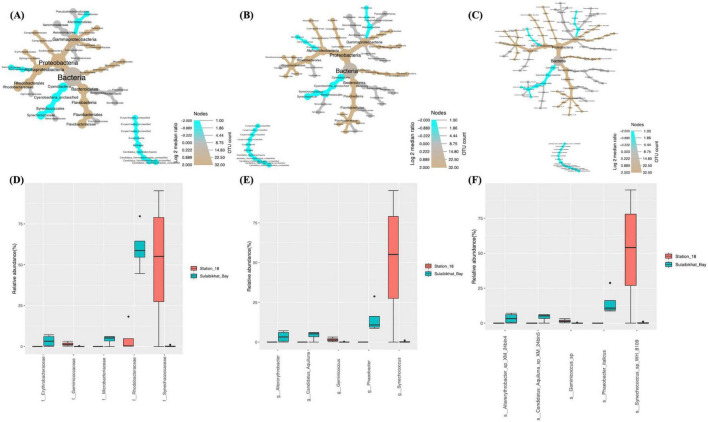
Comparison of microbial abundances between St. MPA-2 in Sulaibikhat Bay and southern offshore waters (St. 18). Differential heat trees were generated at the **(A)** family, **(B)** genus, and **(C)** species levels. The size and color of the nodes are mapped relative to the observed read numbers and the log 2 median ratio, respectively. Differential abundance analysis was performed at the **(D)** family, **(E)** genus, and **(F)** species levels comparing St. MPA-2 in Sulaibikhat Bay (blue) with southern offshore waters (St. 18; red). All taxa depicted on the graphs have a *q*-value < 0.05 and a *p*-value < 0.05 (based on non-parametric *t*-test).

Differential abundance analyses revealed that, compared to St. K6 and St. A, St. 18 had the most significant difference in the microbiome compared to the St. MPA-2 in Sulaibikhat Bay. The abundance of two bacterial phyla, Bacteroidetes (*p* = 0.021) and Cyanobacteria (*p* = 0.0265), exhibited significant differences between the two locations. Within the phylum Bacteroidetes, the class Flavobacteriia, order Flavobacteriales, family *Flavobacteriaceae*, genus *Phaeobacter*, and species *Phaeobacter _italicus* exhibited significantly preferential abundance (*p* = 0.021) at St. MPA-2. Within the phylum Cyanobacteria, the class Cyanobacteria_unclassified, order Synechococcales, family *Synechococcaceae*, genus *Synechococcus*, and species *Synechococcus_sp_WH_8109* were significantly preferentially abundant (*p* = 0.0265) at St. 18. The order Rhodobacterales and family *Rhodobacteraceae* also exhibited significantly higher abundance (*p* = 0.029) at St. MPA-2 (Figures 5A–C and Supplementary Table 7). The variance in abundances of the collective bacterial and archaeal kingdoms themselves borders significance between the two stations (St. MPA-2 and St. 18; *p* = 0.05).

Results from differential abundance analysis revealed that the difference in bacterial and archaeal communities between St. 18 and St. MPA-2 was starker than either the difference between St. A and St. MPA-2 or between St. K6 and St. MPA-2. Seven of 23 families detected were significantly differentially abundant between the two stations. Four of these families, *Rhodobacteraceae* (*p* = 0), *Microbacteriaceae* (*p* = 0.001), *Erythrobacteraceae* (*p* = 0.022), and *Halieaceae* (*p* = 0.04), had significantly higher abundances at St. MPA-2. The remaining three families, *Synechococcaceae* (*p* = 0.0096), *Geminicoccaceae* (*p* = 0.016), and *Alteromonadaceae* (*p* = 0.037), were significantly more abundant at St. 18 (Figure 5D and Supplementary Table 10).

Abundance of eight out of 31 genera was significantly different between the two stations. Specifically, the five genera *Phaeobacter* (*p* = 0), *Candidatus_Aquiluna* (p = 0.0007), *Altererythrobacter* (*p* = 0.014), *Dokdonia* (*p* = 0.021), and *Halieaceae_unclassified* (*p* = 0.038) were significantly more abundant in St. MPA-2. The genera *Synechococcus* (*p* = 0.006), *Geminicoccus* (*p* = 0.01), and *Alteromonas* (*p* = 0.02) were significantly more abundant in St. 18 (Figure 5E and Supplementary Table 10).

Nine out of 38 taxa detected at the species level showed significantly differential abundance between the two stations. The species *Phaeobacter_italicus* (*p* = 0), *Candidatus_Aquiluna_sp_XM_24bin5* (*p* = 0.0008), *Altererythrobacter_sp_XM_24bin4* (*p* = 0.012), *Dokdonia_ donghaensis* (*p* = 0.019), and *Gamma_proteobacterium_IMCC3088* (*p* = 0.046) were significantly more abundant at St. MPA-2. Four species, *Synechococcus_sp_WH_8109* (*p* = 0.006), *Geminicoccus_sp* (*p* = 0.009), *Alteromonas_macleodii* (*p* = 0.018), and *Synechococcus_wp_RS9916* (*p* = 0.034), were significantly more abundant in St. 18 (Figure 5F and Supplementary Table 10).

### 3.5 Multivariate analysis between environmental and biological variables

#### 3.5.1 Microbiome: spatial differences

The pseudo-t-statistics and the associated permutational (perm) and Monte Carlo (MC)-based significance tests in PERMANOVA suggested significant differences in microbiome across all taxonomic levels examined between St. MPA-2 and the other sampling locations (Table 2). The permutation *p*-values should be given preference over the Monte-Carlo *p*-values when the number of unique permutations is large (e.g., 100 or more). Conversely, when the number of possible permutations is limited, the Monte-Carlo *p*-value should be used instead. In this analysis, the number of possible unique permutations was 35. Therefore, Monte-Carlo *p*-values were given preference over Permutational *p*-values as a confirmatory measure of significance for the between group differences. The PERMANOVA test also revealed the dissimilarity (%) between and within the sampled areas. St. MPA-2 had the highest within-group dissimilarity (56.2%) compared to other areas (44.4–53.6%). Nevertheless, the higher between-group (69.2–83.5%) to within-group dissimilarity at St. MPA-2 made this location significantly different from the others.

**TABLE 2:** PERMANOVA test showing the extent of spatial differences in the bacterial community structure between the sampling locations tested.

Areas - pair-wise test	Species-level		Genus-level		Family-level		Unique perms.
	**Pseudo-t**	***P*(MC)**	**Pseudo-t**	***P*(MC)**	**Pseudo-t**	***P*(MC)**	
St. MPA-2 versus St. K6	2.110	***0.027***	2.131	***0.026***	2.280	***0.029***	35
St. MPA-2 versus St. A	2.107	***0.035***	2.152	***0.024***	2.269	***0.023***	35
St. MPA-2 versus St. 18	2.642	***0.011***	2.763	***0.005***	2.952	***0.004***	35
St. K6 versus St. A	0.969	0.455	0.937	0.457	0.836	0.553	35
St. K6 versus St. 18	1.414	0.136	1.404	0.16	1.371	0.164	35
St. A versus St. 18	1.104	0.302	1.062	0.356	0.940	0.435	35

Significant differences are marked in bold italics.

**FIGURE 6:**
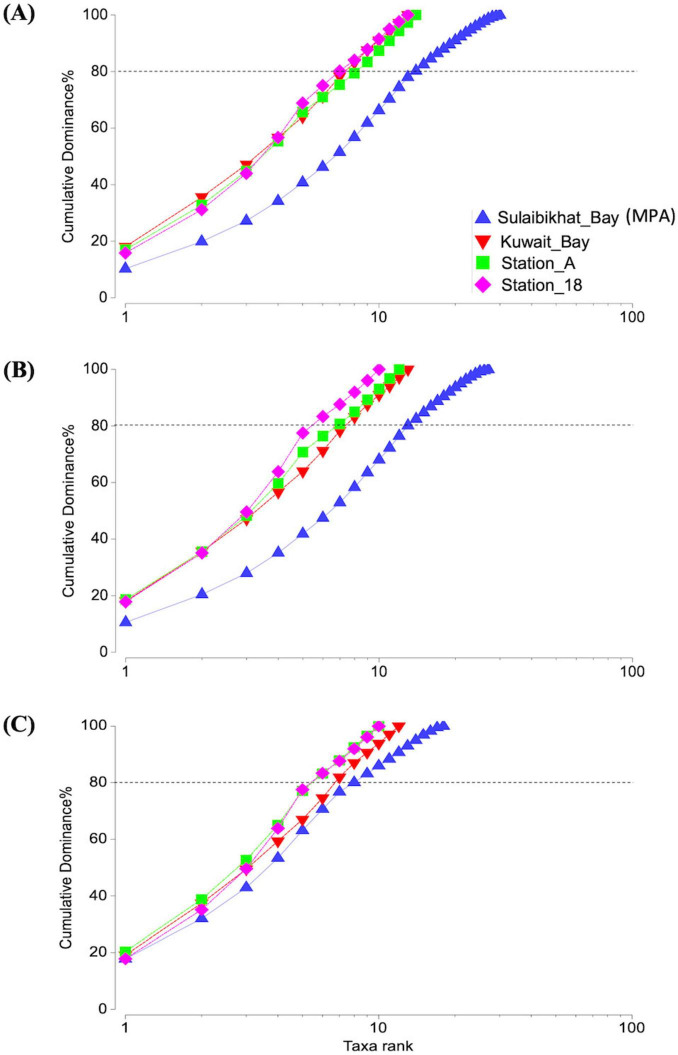
Cumulative k-dominance plot showing the diversity and dominance of taxa at each taxonomic level. Plots depicting the contribution of different bacterial taxa to average OTU density per sample (i.e., OTU per liter) in each sampling station at the **(A)** species, **(B)** genus, and **(C)** family levels. The horizontal dotted line marks the number of taxa cumulatively contributing to 80% of total OTU density.

Furthermore, PCA revealed no overlapping clusters between St. MPA-2 and St. K6, between St. MPA-2 and St. A, or between St. MPA-2 and St. 18. Collectively, these findings indicate the presence of two distinct microbiomes between each pair of stations (Supplementary Figures 2C, E and Supplementary Table 11). These finding were further corroborated by the ANOSIM results comparing each pair of stations: St. MPA-2 versus St. K6 (*R* = 0.51; *p* = 0.05) (Supplementary Figure 2B), St. MPA-2 versus St. A (*R* = 0.823; *p* = 0.024) (Supplementary Figure 2D), and St. MPA-2 versus St. 18 (*R* = 0.948; *p* = 0.03) (Supplementary Figure 2F). The Cumulative k-dominance plot revealed the diversity and dominance of bacterial taxa at the family, genus, and species levels. At all taxonomic levels analyzed, St. MPA-2 was more diverse (low dominance), and St. 18 was less diverse (high dominance). At the species level, 14 species cumulatively contributed 80% of the total bacterial abundance in St. MPA-2. Conversely, only seven species comprised 80% of the average bacterial abundance in St. 18. At the genus and family levels, these numbers came down to 13 and 8 in St. MPA-2, and five St. 18 (Figure 6).

#### 3.5.2 Microbial assemblage patterns

**FIGURE 7:**
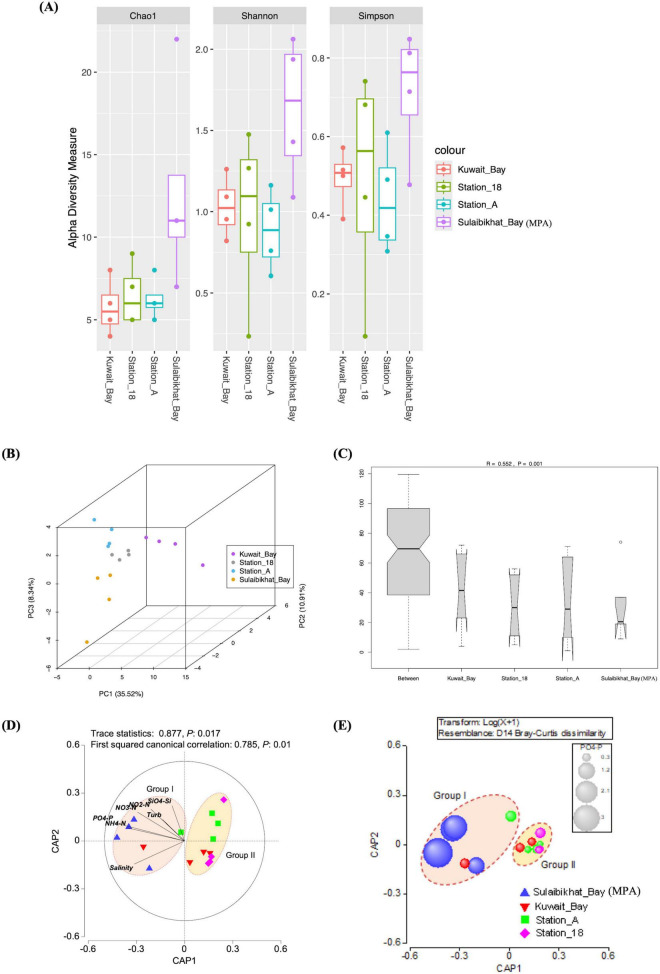
The microbial community in St. MPA-2 in Sulaibikhat Bay is significantly different than that of surrounding waters and is most significantly affected by levels of inorganic phosphate. **(A)** Three alpha diversity indices (Chao1, Shannon, and Simpson) were used to describe the diversity of bacterial and archaeal communities in St. MPA-2 in Sulaibikhat Bay (*n* = 4) and surrounding waters. Diversity was assessed in samples collected between November 2019 and February 2020. Boxplots show the 25th percentile, median, and 75th percentile. The whiskers are defined as: upper whisker = min(max(x), Q_3 + 1.5 × IQR); lower whisker = max(min(x), Q_1 – 1.5 × IQR). **(B)** Beta diversity analyses, the dots on the PCA plot represent single samples. The locations are color coded, an index to which has been provided on the right-hand side panel. **(C)** Analysis of similarity (ANOSIM) analysis plots. R2 denotes the similarity coefficient at a confidence interval of 99.95% (*p* < 0.05). **(D)** Genus-level CAP analysis showing grouping of sampling locations in the coastal and offshore waters of Kuwait during the fall-winter period (November 2019-February 2020). Environmental variables with a Pearson correlation of >0.2 with the CAP axes were overlaid as vectors. **(E)** Bubble plot showing environmental variable PO_4_-P (μM) overlaid on the CAP ordination plots for the bacterial community species-level data. The bubble sizes are proportional to the values of each environmental variable. The bubble colors represent different areas from where samples were collected.

Alpha diversity analysis was performed to assess and compare the number of different taxa documented, the species richness, and species distribution between St. MPA-2 and the other locations (St. K6, St. A, and St. 18) (Figure 7A). The Shannon and Simpson indices comparing St. MPA-2 and St. K6 revealed that microbial diversity levels were borderline significantly different between the two locations (*p* = 0.05; Supplementary Figure 3A and Supplementary Table 12). St. MPA-2 consistently demonstrated significantly greater bacterial and archaeal diversity than St. K6.

St. A, which is located in northern coastal waters to the northeast of Kuwait Bay, has significantly less species richness and diversity compared to St. MPA-2 in Sulaibikhat Bay [Shannon index (*p* = 0.028), Simpson index (*p* = 0.045)] (Supplementary Figure 3B and Supplementary Table 12).

No significant difference in species richness or evenness was observed between St. 18 and St. MPA-2 in Sulaibikhat Bay. St. 18 is located in the southern offshore waters southeast of Kuwait Bay. It is the most remote of the four stations analyzed and the least subject to anthropogenic influence (Supplementary Figure 3C and Supplementary Table 12).

The *Pi* statistics in the SIMPROF test suggested significant clustering of the sampling locations into two groups (*Pi*: 6.92, Sig (%): 0.3). Group I was dominated by all MPA samples, the fall (November) samples from Kuwait Bay (St. K6), and the area near the northern coastal waters (St. A), and Group II by all other samples. The pseudo-t statistics embedded in the PERMANOVA test also supported significant disparity between the groups (Pseudo t: 3.198, p (perm) = 0.001, p (MC) = 0.001).

Overall, PCA (Figure 7B), ANOSIM (Figure 7C), and PERMANOVA analyses revealed that the microbiome in the MPA is significantly different from the microbiome in the surrounding waters. Furthermore, the CAP analysis revealed the importance of spatial and seasonal environmental gradients influencing the microbial communities in the northwest Arabian Gulf (Figure 7D and Supplementary Figure 4). The size of the first squared canonical correlations was large (δ1^2^: 0.783–0.785), indicating the strength of the association between the multivariate environmental data cloud and grouping of samples as observed in the SIMPROF analysis. The role of each environmental variable on the bacterial assemblage patterns was visualized by a vector overlay using Pearson correlations of >0.2. The first canonical axis significantly separated Group I samples (representing St. MPA-2) from Group II (collectively representing stations K6, A, and 18) at all taxonomic levels.

The Bubble plot with environmental variables overlaid on the CAP ordination plot showed that in Group I, constituted mainly by samples from the MPA, dissolved inorganic phosphorous and nitrogen levels were high and silicic acid low. Water temperature and salinity levels were not significantly different between Groups I and II (Figure 7E and Supplementary Figure 5).

**FIGURE 8:**
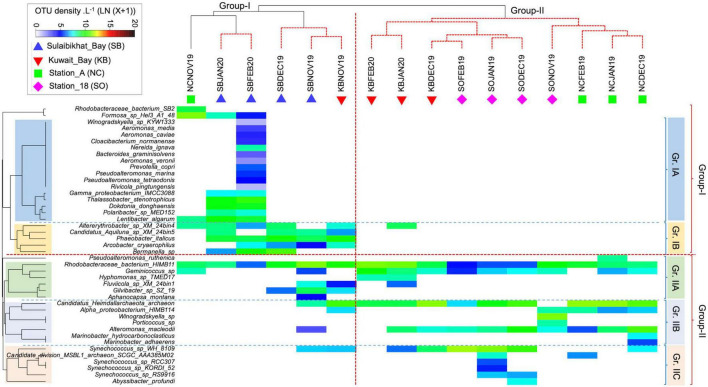
Matrix analysis of the microbial OTU abundance data. Interactive heatmap shows the species and sample association patterns during fall-winter periods in the coastal and offshore waters of Kuwait in the northern Arabian Gulf. NC, northern coastal; SO, southern offshore.

The interactive heatmap visualized the species-sample association patterns inferred from the bacterial OTU counts (i.e., abundances) from different sampling areas, with the color intensity indicating the log-transformed (LN) abundance. This provides compelling evidence of how the bacterial assemblage patterns shaped the clustering of sampling locations (as evident in the CAP analysis of species-level data) (Figure 8). The vertical dotted red line in the figure indicates a significant difference in bacterial communities between the two main sample groups. The clustering may reflect underlying environmental gradients such as salinity, water temperature, nutrients, turbidity, etc., discriminating the sample groups. On the other hand, the horizontal dotted lines divided the bacterial species into two main assemblages (Group I and Group II), which were further subdivided into Gr. IA, IB, Gr. IIA, IIB, and IIC. While the Group-I species were mainly responsible for the Group-I sample assemblage, the Group-II species were mainly responsible for the Group-II sample assemblage pattern. This observation suggests that fall-winter environmental conditions in St. MPA-2 in Sulaibikhat Bay (sample Group I) supported Group I species over Group II.

In the Group I species assemblage, with subdivisions Gr. IA and Gr. IB, IA species in general, and those unique to the MPA in particular, were more prevalent at the peak of winter in January and February, while IB species appeared in both fall and winter periods. The fact that Gr.1A species were absent in fall and early winter in the same area points to their preference for colder temperatures.

The change in environmental conditions from November (fall) through February (peak of winter) supported a consistent increase in bacterial diversity and abundance in the MPA. Such a seasonal pattern was not apparent in other sampling areas. The pattern in the Group-II species cluster was similar, with the IIA, IIB, and IIC species each showing a preference for different locations (Figure 8).

**FIGURE 9:**
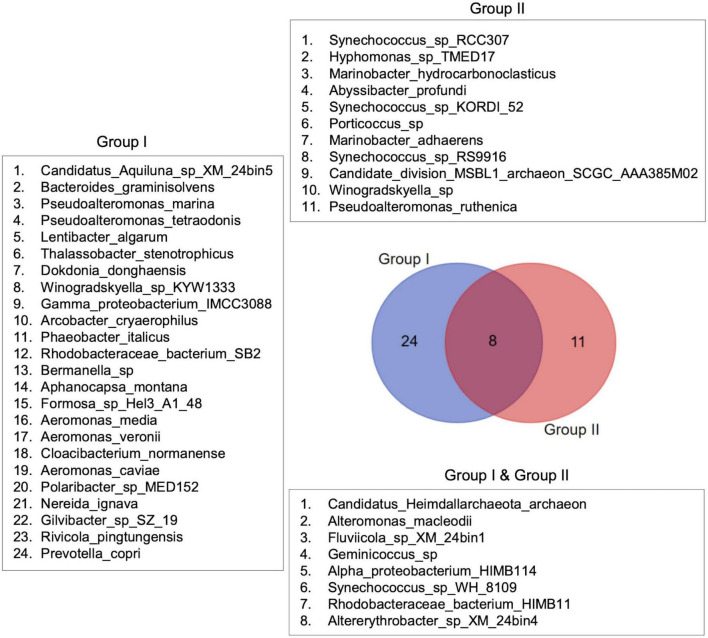
Species distribution patterns in the sample groups. Species distribution patterns are shown in the form of a Venn diagram. The diagram depicts the difference in bacterial species composition between the sample groups identified through multivariate analyses.

The species distribution pattern between the sample groups revealed low taxa turnover (i.e., high beta diversity) between Group I and Group II. Out of 43 species reported in this study, 24 were found exclusively in Group I, dominated by MPA samples, and 11 were found only in Group II samples. Eight species occurred commonly in both groups (Figure 9). Thus, the analysis supports the interactive heatmap findings that the bacterial community in St. MPA-2 is significantly different from the rest of the coastal and offshore locations sampled during this study.

#### 3.5.3 Biotic-environmental interactions

The Spearman rank correlation (Rho) based analysis of the biotic and environmental resemblance matrices suggested significant relationships (*p* < 0.05) between the bacterial community structure and the physicochemical environment. The rank correlation between the two matrices provided a valuable output on how closely the environmental variables captured the variability in bacterial and archaeal taxa on a scale from zero to one. In this study, the environmental correlation with different taxonomic levels was close, with Rho values ranging from 0.498 to 0.511 (*p* = 0.0001–0.001).

Additionally, the biotic-environmental interaction analysis for the best suit of environmental variables provided the Global test statistic (Rho, Spearman correlation) and the test significance level at each taxonomic level (species, genus, and family). As Rho reflects the correlation between the environmental variables and the biotic data, the analysis indicated how well a combination of environmental variables explained the variation in the microbiome at different taxonomic levels. The highest correlation (Rho = 0.739) was observed with phosphate (PO_4_-P) alone for the species-level data. The multi-variable combinations showed slightly lower correlations than the single-variable PO_4_-P, with turbidity and nitrogenous nutrients (NO_2_-N, NO_3_-N, NH_4_-N) being common factors in the combinations. For the genus-level data, phosphate (PO_4_-P) alone showed the highest correlation (Rho = 0.749). The combinations with five variables also had a relatively high correlation with the addition of water temperature and turbidity. Combinations of three and four variables showed slightly lower correlations (0.685 and 0.678, respectively), with turbidity, PO_4_-P, and NH_4_-N being common in both. The five-variable combinations provided the lowest correlations but consistently included water temperature, turbidity, and PO_4_-P and NH_4_-N. As with species and genus-level data sets, the family-level data also showed the highest correlation (Rho = 0.711) with PO_4_-P alone. The best five-variable combination, with a correlation of 0.669, includes water temperature, salinity, NO_3_-N, PO_4_-P, and NH_4_-N (Table 3).

**TABLE 3:** The biotic-environmental interaction analysis procedure (BIOENV) with the five best sets of environmental variables (single or combined) shows the maximum correlation with the bacterial community patterns in Kuwaiti waters during the fall-winter period.

**Species-level data** Global test Sample statistic (Rho): 0.739 Significance level of sample statistic: 0.1%		
*Best results* No. Vars	Corr.	Selections
1	0.739	PO_4_-P
3	0.701	Turbidity, PO_4_-P, NH_4_-N
4	0.689	Turbidity, NO_2_-N, PO_4_-P, NH_4_-N
5	0.689	Water temperature, Turbidity, NO_3_-N, PO_4_-P, NH_4_-N
4	0.689	Water temperature, Turbidity, PO_4_-P, NH_4_-N
**Genus-level data** Global test Sample statistic (Rho): 0.749 Significance level of sample statistic: 0.03%		
*Best results* No.Vars	Corr.	Selections
1	0.749	PO_4_-P
3	0.685	Turbidity, PO_4_-P, NH_4_-N
4	0.678	Turbidity, NO_2_-N, PO_4_-P, NH_4_-N
5	0.675	Water temperature, Turbidity, NO_3_-N, PO_4_-P, NH_4_-N
4	0.675	Water temperature, Turbidity, PO_4_-P, NH_4_-N
**Family-level data** Global test Sample statistic (Rho): 0.711 Significance level of sample statistic: 0.1%		
*Best results* No.Vars	Corr.	Selections
1	0.711	PO_4_-P
5	0.669	Water temperature, Salinity, NO_3_-N, PO_4_-P, NH_4_-N
5	0.661	Salinity, NO_3_-N, NO_2_-N, PO_4_-P, NH_4_-N
4	0.655	Water temperature, NO_3_-N, PO_4_-P, NH_4_-N
4	0.651	Salinity, NO_2_-N, PO_4_-P, NH_4_-N

Thus, PO_4_-P appears to be the single most influential environmental variable affecting microbial life across all taxonomic levels analyzed, suggesting that phosphate concentrations significantly shape the bacterial community structure in the Kuwaiti coastal waters of the Arabian Gulf. PO_4_-P, water temperature, and turbidity were the environmental variables that consistently showed high correlations at all taxonomic levels analyzed and appeared in the “Best results” multiple times. Ammonium (NH_4_-N) and nitrate (NO_2_-N, NO_3_-N) were also recurrent, although their correlations were not as strong as those of phosphate. It is noticeable that salinity became a more significant factor at the family level compared to the genus and species levels. Thus, the BIOENV analysis showed that each environmental variable collected in this study contributed to the overall microbial community patterns, although their relative influence varied across the taxonomic levels.

The Biotic-Environmental interaction analysis between species present exclusively in Group I (24 species) and Group II (11 species) with the corresponding environmental data revealed their differential environmental preferences that go well with the nutrient cycling and plankton nutrient assimilation strategies. In Group I stations, where fresh inorganic nitrogen and phosphorous availability was higher due to higher anthropogenic inputs, silicic acid became the single most influential variable affecting the microbiome. Water temperature, salinity, turbidity, nitrate, and silicic acid offered the best combination of variables in Group I (Rho: 0.629). Since Si is the primary substrate for siliceous phytoplankton (diatoms and silico nanoflagellates) that controls their NP assimilation rates, silicic acid in surplus can induce competition between the nano-micro phytoplankton and the resident bacterial community for the available NP resources. Therefore, silicic acid could be an essential variable influencing bacterial community structure in areas with high NP availability. On the other hand, water temperature, salinity, phosphate, nitrite, and ammonium availability proved crucial for the microbiome in other coastal areas and offshore waters (Rho: 0.517). The presence of nitrite and ammonium in the best set of environmental variables suggested the importance of processes like organic matter remineralization, and nitrification/denitrification in regulating the distribution of bacterial and archaeal species endemic to Group II samples (Supplementary Table 13).

## 4 Discussion

The present manuscript reports on the largely unique microbial communities present in the waters of the designated MPA within Sulaibikhat Bay in Kuwait. The Bay is a protected water body under the jurisdiction of Kuwait Environment Public Authority (KEPA). It was established to protect marine biodiversity and to rehabilitate neighboring Kuwait Bay. The health and dynamics of marine ecosystems are greatly affected by microbial life. Advanced molecular research highlighting the distribution, interactions, and adaptations to anthropogenetic and natural stressors of marine microbes are essential for developing a better understanding of water bodies and, by extension, for maintaining the health of these ecosystems (DeLong, 2009). Continual urban development activities near the MPA in Sulaibikhat Bay in Kuwait therefore necessitates the regular surveillance and monitoring of microbial diversity within that water body.

### 4.1 Metagenomic sequencing and assembly

Biodiversity monitoring of Sulaibikhat Bay has been attempted previously, with efforts made to investigate the endemic macrofauna (Al-Zaidan et al., 2003), benthic foraminifera (Al-Enezi and Frontalini, 2015), and higher organisms such as mud skippers (Al-Behbehani and Ebrahim, 2010) within this environment. Microbial communities associated with the micro and macrofauna of Sulaibikhat Bay have been limitedly explored through conventional culture-dependent techniques and microscopic examination (Al-Mohanna et al., 2007; Al-Zaidan et al., 2006). Genome-centric approaches are gaining popularity over such traditional methods for assessment of biodiversity (Doytchinov and Dimov, 2022). Presently high throughput shotgun metagenomic sequencing of DNA isolated from the surface waters of the MPA within Sulaibikhat Bay as well as three other locations distributed throughout neighboring waters in and around Kuwait Bay successfully mapped the bacterial and archaeal communities at these locations. Not surprisingly, bacterial communities were more abundant and diverse compared to archaeal communities. Shotgun metagenomic techniques have been used by numerous research groups for taxonomic and functional annotations of microscopic organisms including bacteria, archaea, viruses, and fungi in aquatic environments (Doytchinov and Dimov, 2022).

### 4.2 Unique microbial communities in the embayment

Relatively high abundances of the bacterial families *Rhodobacteraceae* and *Flavobacteriaceae* were recorded in the surface seawater samples collected from St. MPA-2 in Sulaibikhat Bay. Both these bacterial families were also found in surface waters of Southeast Florida with reported functional roles in hydrolysis of complex molecules, photosynthesis, and degradation of algal products (Campbell et al., 2015). *Rhodobacteraceae* was also the core bacterial family in surface seawater collected from the South China Sea and the coastal waters of Hong Kong and Xiamen (Xia et al., 2021). *Flavobacteriaceae* were exclusively abundant in the warm oligotrophic waters of the South China Sea (Xia et al., 2021). Both these families were associated with *Synechococcus* cultures in the study conducted by Xia et al. (2021). In the present study, the dominant genus within the family *Rhodobacteraceae* that was detected within the samples collected from Kuwaiti waters was *Phaeobacter*, and the dominant species was *P. italicus.* Similarly, the bacterial genera *Cognatishimia and Phaeobacter* from the family *Rhodobacteraceae* were found in the surface waters of Bay of Bengal (Akter et al., 2023).

The Sulaibikhat embayment is considered a pristine habitat, biologically diverse and linked with commercial fishing activities in Kuwait. In the recent past, some signatures of physicochemical stresses such as changing water and sediment profiles, and biological activities were noticed. For example, effluents from nearby desalination plants and the Ghazali outfall are affecting the water column and increasing organic content within the water body. Pyrite deposition in the vicinity of the Ghazali outfall is indicative of sewage accumulation (Alshemmari et al., 2011). In view of such anthropogenically induced eutrophication and the subsequent putative harmful impacts they impart, it is essential to continually biomonitor affected environments, especially protected areas. The relative abundances of the bacterial and archaeal taxa differed between St. MPA-2 and the other sampling locations distributed in and around Kuwait Bay. The variations in the relative abundance of various microbial taxa are likely due to variable environmental and physiochemical conditions during the sampling period, which spanned a period of 4 months.

### 4.3 Spatial variations between the MPA in Sulaibikhat Bay and surrounding waters are attributed to contaminants concentrations

The bacterial richness and distribution recorded within surface waters of St. MPA-2 was significantly greater than those recorded in St. K6, St. A, and St. 18. This is likely attributed to variations in nutrient availability between the locations. Higher levels of inorganic phosphorus and nitrogen were recorded in St. MPA-2 compared to the other locations. The source of these abiotic elements is most likely the effluents received through thirteen discharge points around the Bay (ROPME, 2010). The inorganic and organic load introduced through the effluent ports provide a breeding bed for several microbial communities and likely contribute heavily to the observed richness in distribution and diversity of these communities within these waters. Similarly, greater Shannon and ACE diversity indices were reported for the areas along the tropical seawater habitats near outfalls adjacent to Broward County in the state of Florida (Campbell et al., 2015). Higher Shannon and Faith indices of 18S rRNA operational taxonomic units (eukaryotic microalgae) were also observed at similar locations along Kuwait’s coast (Kumar et al., 2021). Hahnke et al. (2013) reported that bacterial communities are generally more diverse in the phototrophic layers. Sulaibikhat Bay is a shallow, tidal mudflat with a water column spanning ten meters (maximum depth at the Bay entrance). The designated MPA within Sulaibikhat Bay, however, has an average depth of only 2–3 m. The cumulative k-dominance analysis was also suggestive of the dominance of bacterial taxa and increased diversity in St. MPA-2 compared to the other locations distributed throughout the surrounding waters (St. K6 in Kuwait Bay, St. A in northern coastal waters, and St. 18 in southern offshore waters).

Additionally, differential abundance analysis revealed the taxa that are overrepresented in St. MPA-2 as compared to the other three stations. The species *Phaeobacter_italicus*, family *Rhodobacteraceae*, and order Rhodobacterales were more abundant in St. MPA-2 (*p* < 0.05) versus in St. K6, St. 18, and St. A, respectively. These microbes play a role in carbon assimilation and hence their enrichment in eutrophic marine environments is not surprising (Campbell et al., 2015). *Rhodobacteraceae* is a group of bacteria that exist in marine environments and are functionally involved in biogeochemical cycling of elements such as sulfur and are known to establish symbiotic relationships with other aquatic microorganisms (Pujalte et al., 2014). The species assemblage analysis performed in this study corroborates the differential abundance findings. Namely, 24 species were found exclusively in Group I (St. MPA-2) and 11 species were specific to Group II (St. A, St. 18, and St. K6; Figure 9).

### 4.4 Multivariate analysis between environmental and biological variables with emphasis on phosphate as the dominating factor

The microbial populations detected in St. MPA-2 present as a distinct group (Group I) on the canonical analysis plots. The other three stations in Kuwait Bay and surrounding waters collectively formed a separate cluster (Group II). Similar clustering was observed through PCA. The spatial distance and environmental gradients between the MPA and other station locations seems to be the driving factor for the observed clustering pattern. Station K6, located within Kuwait Bay, is the most proximal, whereas St. A is located northeast of the Bay between the Kuwaiti coastline and the Kuwaiti island of Bubiyan, and Station 18 is located in offshore waters southeast of the Bay and is the most remote and distant (Fakhraldeen et al., 2023). These findings were further substantiated by the interactive heatmap clustering. In addition to the spatial differences, varying environmental conditions in the MPA in Sulaibikhat Bay are also speculated to play a role in shaping the microbiome, as environmental parameters such as temperature, salinity, and nutrient (NOx, phosphate, and silicic acid) levels affect the bacterial community structure, as reported from the southern coastal waters of Korea (Cui et al., 2019). In the present study, a high correlation between the bacterial taxa and phosphate levels (Rho > 0.700) suggests this inorganic nutrient significantly affects the microbiome in the Sulaibikhat Bay MPA. A study by Xia et al. (2021) reported that the members of the families *Rhodobacteraceae* and *Flavobacteriaceae*, which are abundant in the MPA, are rich in ABC transporters involved in importing nutrients including phosphate, amino acids, and other carbon-containing compounds (Xia et al., 2021). Additionally, members of the family *Microbacteriaceae*, which is significantly enriched in the MPA, have been reported to play significant roles in phosphate solubilization (Rivas et al., 2004). Interestingly, a negative correlation between excess phosphate loading and abundance of members of the family *Flavobacteriaceae* was reported in oligotrophic coastal waters indicating that perhaps the effect of phosphate levels on aquatic microbiomes, at least as they related to the family *Flavobacteriaceae*, may vary in a context specific manner (Chen et al., 2016). Increased phosphorus levels were also shown to shape the microeukaryotic communities of the Pearl River estuary, a bell-shaped estuary at the intersection of the Pearl River and the South China Sea (Zhang et al., 2023).

In this study, the sub-clusters assembled within the main clusters in the heatmap analysis demonstrate the intricate nature of the microbiome at these locations. While the combined effect of various environmental variables can influence the overall biotic structure, the taxa-specific environmental preferences or niches along with seasonal differences in environmental settings at each sampling location, can lead to finer-scale differences in the microbial assemblages. Besides environmental gradients, biotic factors such as competition for limiting nutrient resources by the resident phytoplankton community, and predation (viral lysis and microzooplankton grazing) can also alter the microbiome in the northern Arabian Gulf, which is known for its environmental heterogeneity. This complexity underscores the need for further investigation and understanding of the dynamics of the microbial communities residing in this water body.

## 5 Conclusion

The marine environment of the northwest Arabian Gulf, including Kuwait’s coastline, faces significant natural and anthropogenic stressors. This study focused on microbial communities within Kuwait’s first Marine Protected Area (MPA), established by the United Nations Compensation Commission (UNCC). Surface seawater samples were collected over four consecutive months from four locations (the MPA in Sulaibikhat Bay, Kuwait Bay, northern coastal waters, and southern offshore waters) for microbiome analysis using a shotgun metagenomic sequencing approach. Results revealed significant spatial variability in microbial diversity. The study included alpha and beta diversity analysis, assessment of differences in microbiomes between the study locations, and analysis of microbiome-environmental interactions through multivariate statistical procedures. Results indicated significantly greater microbial diversity within the MPA than in surrounding waters, with dominant families including *Rhodobacteraceae* and *Flavobacteriaceae*. Environmental variables such as water temperature, salinity, and nutrient levels, particularly phosphate levels, significantly influenced the microbiome. These findings underscore the substantial impact of nutrient enrichment and anthropogenic stressors on microbial diversity, highlighting the need for continuous monitoring and targeted conservation efforts to protect the ecological and economic health of the Arabian Gulf marine ecosystem. Future research should prioritize long-term monitoring and the development of strategies to mitigate the impact of anthropogenic activities on vital habitats such as MPAs.

## Data Availability

The datasets presented in this study can be found in online repositories. The names of the repository/repositories and accession number(s) can be found below: https://www.ncbi.nlm.nih.gov/sra/PRJNA945575, Accession numbers: SAMN33788923 - SAMN33788928, https://www.ncbi.nlm.nih.gov/sra/PRJNA910671, Accession numbers: SAMN32137969, SAMN32137971, SAMN32137973, SAMN32137975, SAMN32137977, SAMN32137979, SAMN32137981, SAMN32137983, SAMN32137985, SAMN32137987, SAMN32137989, and SAMN32137991.

## Footnotes

1
https://www.bioinformatics.babraham.ac.uk/projects/fastqc/

2
https://www.rdocumentation.org/packages/stats/versions/3.6.2/topics/prcomp

3
https://bioinformatics.psb.ugent.be/webtools/Venn
