# Supplementary material for: Shotgun metagenomics reveals the interplay between microbiome diversity and environmental gradients in the first marine protected area in the northern Arabian Gulf
Source: Front Microbiol. 2025 Jan 9;15:1479542. doi: 10.3389/fmicb.2024.1479542 (PMC11755137; doi:10.3389/fmicb.2024.1479542)
Supplement: Supplementary file 1 [file Data_Sheet_1.ZIP › MPA_SupplementaryMaterial_Submit_1224/MPA_FigS2.docx]

(a)

(b)

(d)

(c)

(f)

(e)

**Figure S2. Beta** **diversity metrics used to compare community structures between St. MPA-2 in Sulaibikhat Bay and the other sampling stations.** Beta diversity analysis comparing differences between St. MPA-2 (Sulaibikhat Bay) and St. K6 (Kuwait Bay; (a,b)), St. A (Northern Coastal Station (c,d)), and St. 18 (Southern Offshore Station (e,f)) using Principal Component Aanlysis (PCA; a, c, and e) and Analysis of Similarity (ANOSIM; b, d, and f). R and p values are shown.
