# Supplementary material for: Shotgun metagenomics reveals the interplay between microbiome diversity and environmental gradients in the first marine protected area in the northern Arabian Gulf
Source: Front Microbiol. 2025 Jan 9;15:1479542. doi: 10.3389/fmicb.2024.1479542 (PMC11755137; doi:10.3389/fmicb.2024.1479542)
Supplement: Supplementary file 1 [file Data_Sheet_1.ZIP › MPA_SupplementaryMaterial_Submit_1224/MPA_FigS3.docx]

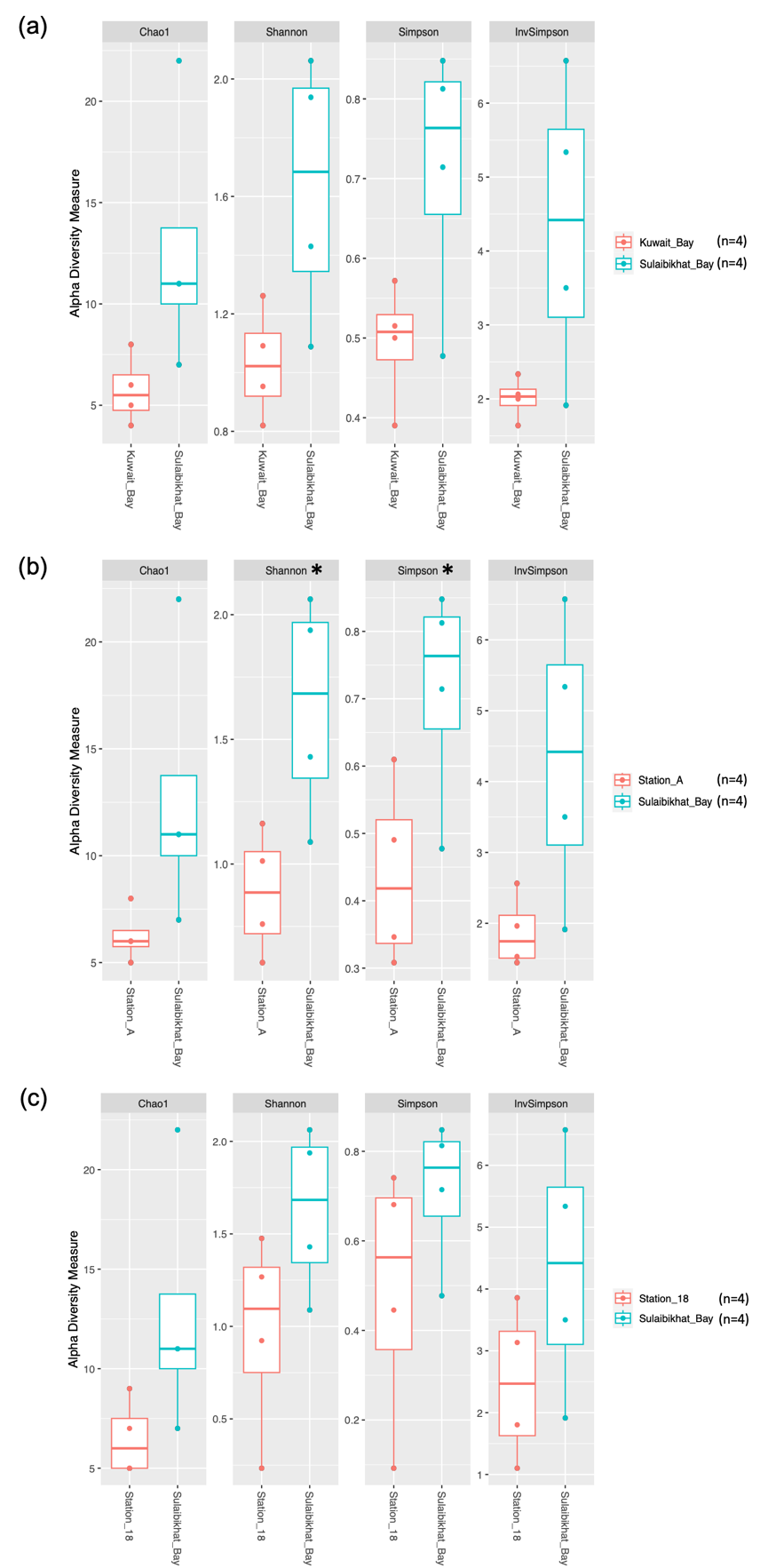


**Figure S3. Alpha diversity analysis – pair-wise station comparisons**. Four alpha diversity metrics (Chao1, Shannon, Simpson, and InvSimpson) were used to describe the diversity of bacterial and archaeal communities in the MPA in Sulaibikhat Bay (n=4) and Station K6 in Kuwait Bay (a)(n=4), St. A in northern coastal waters (b)(n=4), and St. 18 in southern offshore waters (c) (n=4) and compare the levels of diversity between the two stations. Diversity was assessed in samples collected between November 2019 and February 2020. Boxplots show the 25th percentile, median, and 75th percentile. The whiskers are defined as: upper whisker = min(max(x), Q_3 + 1.5 * IQR); lower whisker = max(min(x), Q_1 – 1.5 * IQR). *p<0.05.
