# Supplementary material for: Shotgun metagenomics reveals the interplay between microbiome diversity and environmental gradients in the first marine protected area in the northern Arabian Gulf
Source: Front Microbiol. 2025 Jan 9;15:1479542. doi: 10.3389/fmicb.2024.1479542 (PMC11755137; doi:10.3389/fmicb.2024.1479542)
Supplement: Supplementary file 1 [file Data_Sheet_1.ZIP › MPA_SupplementaryMaterial_Submit_1224/MPA_TableS1.docx]

**Table S1.** Sampling details pertaining to seawater samples collected throughout the study period

| **Sampling date** | **Sampling time** | **GPS coordinates** | **Measured station depth (m)** | **Sample acquisition depth (m)** | **Seawater temperature (^o^C)** | **Salinity** | **Dissolved oxygen levels (ml/L)** |
| --- | --- | --- | --- | --- | --- | --- | --- |
| **Station MPA-2 (Sulaibikhat Bay)** | | | | | | | |
| 10/11/2019 | 10:30am | 29^o^ 20.629N  45^o^ 50.721E | 1.8 | 1.0 | 20.2 | 42.3 | 7.4 |
| 11/12/2019 | 11:23am | 29^o^ 20.646N  47^o^ 50.717E | 2.0 | 1.00 | 18.37 | 42.11 | 7.32 |
| 09/01/2020 | 10:50am | 29^o^ 20.630N  47^o^ 50.702E | 1.7 | 1.02 | 17.37 | 41.78 | 7.67 |
| 06/02/2020 | 9:50am | 29^o^ 20.621N  47^o^ 50.698E | 1.4 | 1.06 | 18.57 | 42.47 | 7.3 |
| 08/03/2020 | 10:55am | 29^o^ 20.626N  47^o^ 50.709E | 1.9 | 1.01 | 20.29 | 41.09 | 7.70 |
| 15/11/2020 | 10:48am | 29^o^ 20.621N  47^o^ 50.698E | 2.0 | 0.99 | 22.84 | 43.24 | 5.17 |
| **Station K6 (KuwaitBay)** | | | | | | | |
| 12/11/2019 | 9:10am | 29o 26.987N  47o 58.009E | 11.0 | 1.20 | 24.41 | 41.93 | 6.73 |
| 05/12/2019 | 9:00am | 29o 26.987N  47o 57.995 | 10.7 | 1.38 | 20.51 | 40.56 | 8.02 |
| 05/01/2020 | 9:25am | 29o 26.997N  47o 57.986E | 10.7 | 1.22 | 17.13 | 40.27 | 7.41 |
| 03/02/2020 | 8:45am | 29o 26.998N  47o 58.007E | 10.5 | 1.31 | 14.83 | 40.34 | 8.17 |
| **Station A (Northern Coastal Station)** | | | | | | | |
| 11/11/2019 | 8:40am | 29o 35.995N  48o 09.997E | 6.8 | 1.3 | 22.12 | 42.5 | 6.67 |
| 04/12/2019 | 8:45am | 29o 35.995N  48o 10.004E | 6.1 | 1.31 | 19.55 | 40.4 | 7.2 |
| 07/01/2020 | 8:30am | 29o 35.996N  48o 10.002E | 6.7 | 1.29 | 15.76 | 38.0 | 8.55 |
| 02/02/2020 | 9:25am | 29o 35.995N  48o 10.001E | 6.5 | 1.36 | 13.96 | 35.4 | 8.81 |
| **Station 18 (Southern Offshore Station)** | | | | | | | |
| 03/11/2019 | 9:15am | 29o 03.727N  48o 30.336E | 20.3 | 1.39 | 28.24 | 40.84 | 5.67 |
| 01/12/2019 | 9:25am | 29o 03.726N  48o 30.335E | 19.8 | 1.29 | 24.21 | 40.64 | 6.86 |
| 06/01/2020 | 8:45am | 29o 03.739N  48o 30.340E | 21.0 | 1.31 | 19.79 | 40.79 | 7.23 |
| 04/02/2020 | 8:50am | 29o 03.726N  48o 30.332E | 21.1 | 1.31 | 16.28 | 40.08 | 7.61 |
