# Supplementary material for: Shotgun metagenomics reveals the interplay between microbiome diversity and environmental gradients in the first marine protected area in the northern Arabian Gulf
Source: Front Microbiol. 2025 Jan 9;15:1479542. doi: 10.3389/fmicb.2024.1479542 (PMC11755137; doi:10.3389/fmicb.2024.1479542)
Supplement: Supplementary file 1 [file Data_Sheet_1.ZIP › MPA_SupplementaryMaterial_Submit_1224/MPA_FigS4.docx]

(MPA)

**Figure S4. Canonical Analysis of Principal Coordinates (CAP) of bacterial OTU counts at various taxonomic levels.** CAP analysis showing grouping of sampling locations in the coastal and offshore waters of Kuwait during the fall-winter period (Nov 2019-Feb 2020) at the (A) species, (B) genus, and (C) family levels. Environmental variables with a Pearson correlation of >0.2 with the CAP axes were overlaid as vectors. Results from the Permutation test are also provided.
