# Supplementary material for: Shotgun metagenomics reveals the interplay between microbiome diversity and environmental gradients in the first marine protected area in the northern Arabian Gulf
Source: Front Microbiol. 2025 Jan 9;15:1479542. doi: 10.3389/fmicb.2024.1479542 (PMC11755137; doi:10.3389/fmicb.2024.1479542)
Supplement: Supplementary file 1 [file Data_Sheet_1.ZIP › MPA_SupplementaryMaterial_Submit_1224/MPA_FigS5.docx]

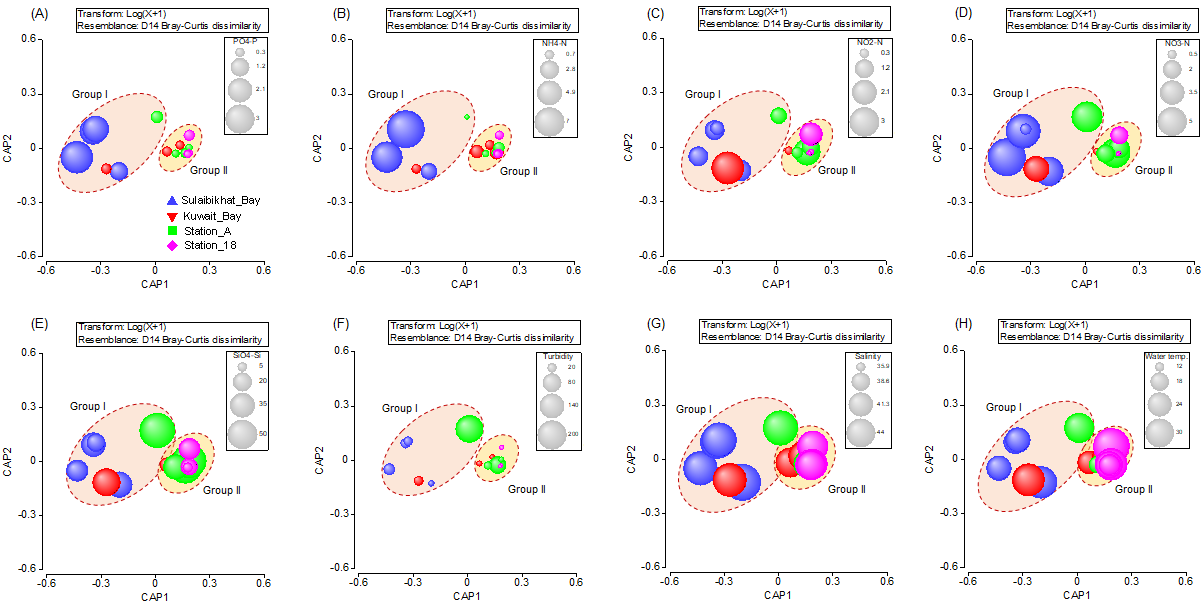


(MPA)

**Figure S5.** **Bubble plots showing environmental variables overlaid on the CAP ordination plot for the bacterial community species-level data.** The bubble sizes are proportional to the values of each environmental variable. The bubble colors represent different areas from where samples were collected. (A) PO_4_-P (µM) (B) NH_4_-N (µM) (C) NO_2_-N (µM) (D) NO_3_-N (µM) (E) SiO_4_-Si (µM) (F) Turbidity (NTU) (G) Salinity (H) Water temperature (⁰C)
