# Supplementary material for: Shotgun metagenomics reveals the interplay between microbiome diversity and environmental gradients in the first marine protected area in the northern Arabian Gulf
Source: Front Microbiol. 2025 Jan 9;15:1479542. doi: 10.3389/fmicb.2024.1479542 (PMC11755137; doi:10.3389/fmicb.2024.1479542)
Supplement: Supplementary file 1 [file Data_Sheet_1.ZIP › MPA_SupplementaryMaterial_Submit_1224/MPA_TableS12.docx]

| **Alpha diversity measure** | **St. MPA-2 versus St. K6** | **St. MPA-2 versus St. A** | **St. MPA-2 versus St. 18** |
| --- | --- | --- | --- |
| Chao1 | 0.08062373 | 0.09520183 | 0.11249861 |
| Shannon | 0.0505899 | 0.02801458 | 0.11386404 |
| Simpson | 0.05439201 | 0.04469345 | 0.23502661 |
| InvSimpson | 0.06577557 | 0.0588074 | 0.17251896 |

**Table S24.** Alpha diversity metrics – pair-wise station comparisons.

| **Location** | **mean** | **std** | **r** | **Min** | **Max** | **Q25** | **Q50** | **Q75** | **Index** |
| --- | --- | --- | --- | --- | --- | --- | --- | --- | --- |
| Sulaibikhat_Bay | 12.75 | 6.44851404 | 4 | 7 | 22 | 10 | 11 | 13.75 | Chao1 |
| Sulaibikhat_Bay | 1.62953049 | 0.45265319 | 4 | 1.0884906 | 2.06213741 | 1.34450601 | 1.68374698 | 1.96877146 | Shannon |
| Sulaibikhat_Bay | 0.71308324 | 0.16694201 | 4 | 0.47743872 | 0.8478713 | 0.65514877 | 0.76351146 | 0.82144593 | Simpson |
| Sulaibikhat_Bay | 4.33137538 | 2.04718614 | 4 | 1.91365117 | 6.57338168 | 3.10432952 | 4.41923434 | 5.64628021 | InvSimpson |
| Kuwait_Bay | 5.75 | 1.70782513 | 4 | 4 | 8 | 4.75 | 5.5 | 6.5 | Chao1 |
| Kuwait_Bay | 1.03139247 | 0.18925176 | 4 | 0.81980839 | 1.26150082 | 0.91969824 | 1.02213034 | 1.13382458 | Shannon |
| Kuwait_Bay | 0.49436555 | 0.07595076 | 4 | 0.39024787 | 0.57180652 | 0.47272408 | 0.5077039 | 0.52934536 | Simpson |
| Kuwait_Bay | 2.00973481 | 0.28614099 | 4 | 1.64001066 | 2.33539287 | 1.91065141 | 2.03176786 | 2.13085126 | InvSimpson |
| Station_A | 6.25 | 1.25830574 | 4 | 5 | 8 | 5.75 | 6 | 6.5 | Chao1 |
| Station_A | 0.8846947 | 0.24986186 | 4 | 0.60482512 | 1.16217513 | 0.72080292 | 0.88588928 | 1.04978106 | Shannon |
| Station_A | 0.43870815 | 0.13850375 | 4 | 0.30821094 | 0.6098579 | 0.33675122 | 0.41838188 | 0.52033881 | Simpson |
| Station_A | 1.87526801 | 0.51152284 | 4 | 1.44552734 | 2.56316862 | 1.50863506 | 1.74618804 | 2.11282099 | InvSimpson |
| Station_18 | 6.5 | 1.91485422 | 4 | 5 | 9 | 5 | 6 | 7.5 | Chao1 |
| Station_18 | 0.97489019 | 0.54426594 | 4 | 0.2334756 | 1.47555063 | 0.75063361 | 1.09526726 | 1.31952383 | Shannon |
| Station_18 | 0.48991501 | 0.29411358 | 4 | 0.09232742 | 0.74082806 | 0.35722718 | 0.56325229 | 0.69594012 | Simpson |
| Station_18 | 2.47456269 | 1.24976594 | 4 | 1.10171886 | 3.85844237 | 1.6280657 | 2.46904476 | 3.31554175 | InvSimpson |
