# Supplementary material for: Shotgun metagenomics reveals the interplay between microbiome diversity and environmental gradients in the first marine protected area in the northern Arabian Gulf
Source: Front Microbiol. 2025 Jan 9;15:1479542. doi: 10.3389/fmicb.2024.1479542 (PMC11755137; doi:10.3389/fmicb.2024.1479542)
Supplement: Supplementary file 1 [file Data_Sheet_1.ZIP › MPA_SupplementaryMaterial_Submit_1224/MPA_TableS13.docx]

**Table S25. The biotic-environmental interaction analysis procedure (BIOENV) results.** BEST analysis was performed with the five best sets of environmental variables (single or combined) that shows the maximum correlation with the bacterial community patterns at the species-level in Group I and Group II sample clusters, as identified by the CAP ordination.

**Group I**

*Best results*

*The single most variable*

No.Vars Corr. Selections

1 0.461 SiO_4_-Si

*Variable combination*

No.Vars Corr. Selections

5 0.629 Water temperature, Salinity, Turbidity, NO_3_-N, SiO_4_-Si

4 0.621 Water temperature, Turbidity, NO_2_-N, SiO_4_-N

4 0.618 Water temperature, Turbidity, NO_3_-N, NO_2_-N

5 0.618 Water temperature, Salinity, Turbidity, NO_3_-N, NO_2_-N

5 0.604 Water temperature, Salinity, Turbidity, NO_2_-N, SiO_4_-Si

**Group II**

*Best results*

*The single most variable*

No.Vars Corr. Selections

1 0.357 PO_4_-P

*Variable combination*

No.Vars Corr. Selections

4 0.517 Water temperature, Salinity, NO_2_-N, PO_4_-P

3 0.515 Water temperature, Salinity, NO_2_-N

5 0.507 Water temperature, Salinity, NO_2_-N, PO_4_-P, NH_4_-N

4 0.502 Water temperature, Salinity, NO_2_-N, NH_4_-N

5 0.499 Water temperature, Salinity, NO_3_-N, PO_4_-P, NH_4_-N
