# Supplementary material for: Shotgun metagenomics reveals the interplay between microbiome diversity and environmental gradients in the first marine protected area in the northern Arabian Gulf
Source: Front Microbiol. 2025 Jan 9;15:1479542. doi: 10.3389/fmicb.2024.1479542 (PMC11755137; doi:10.3389/fmicb.2024.1479542)
Supplement: Supplementary file 1 [file Data_Sheet_1.ZIP › MPA_SupplementaryMaterial_Submit_1224/MPA_FigS1.docx]

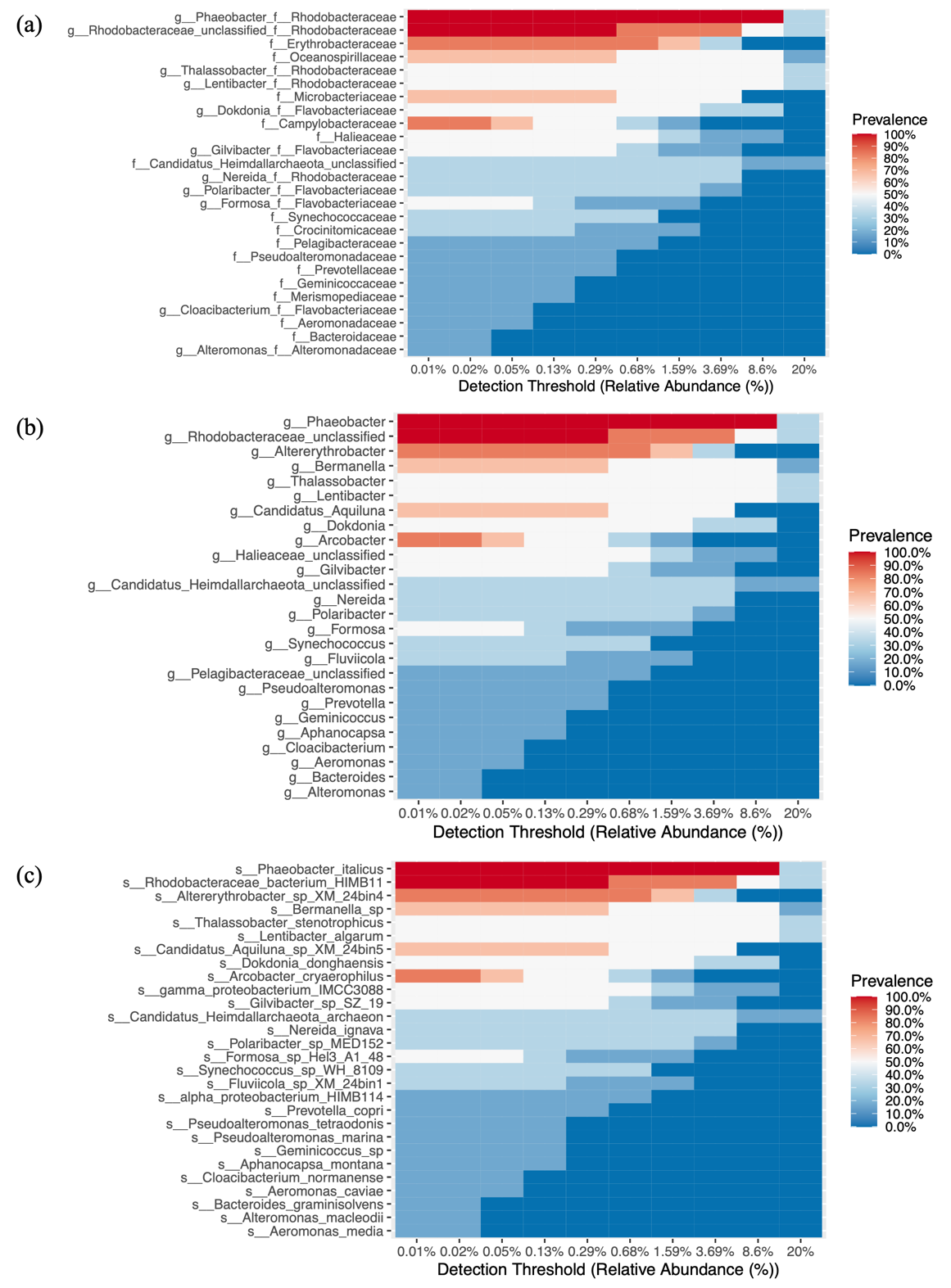


**Figure S1. Description of the core microbiome in St. MPA-2 in Sulaibikhat Bay.** Core microbiome analysis of marine bacteria and archaea was performed on samples from St. MPA-2 (four samples collected monthly between November 2019-February 2020) within the marine protected area in Sulaibikhat Bay at the (a) family, (b) genus, and (c) species levels.
